# The magnitude and temporal changes of response in the placebo arm of surgical randomized controlled trials: a systematic review and meta-analysis

**DOI:** 10.1186/s13063-016-1720-7

**Published:** 2016-12-12

**Authors:** Karolina A. Wartolowska, Benjamin G. Feakins, Gary S. Collins, Jonathan Cook, Andrew Judge, Ines Rombach, Benjamin J. F. Dean, James A. Smith, Andrew J. Carr

**Affiliations:** 1 Oxford NIHR Musculoskeletal Biomedical Research Unit, University of Oxford, Oxford, UK; 2 Botnar Institute of Musculoskeletal Sciences, Nuffield Department of Orthopaedics, Rheumatology and Musculoskeletal Sciences, University of Oxford, Oxford, UK; 3 Nuffield Department of Primary Care Health Sciences, University of Oxford, Oxford, UK; 4 Centre for Statistics in Medicine, University of Oxford, Oxford, UK; 5 MRC Lifecourse Epidemiology Unit, University of Southampton, Oxford, UK

**Keywords:** Surgery, Placebos, Randomized controlled trials, Systematic review, Meta-analysis

## Abstract

**Background:**

Understanding changes in the placebo arm is essential for correct design and interpretation of randomized controlled trials (RCTs). It is assumed that placebo response, defined as the total improvement in the placebo arm of surgical trials, is large; however, its precise magnitude and properties remain to be characterized. To the best of our knowledge, the temporal changes in the placebo arm have not been investigated. The aim of this paper was to determine, in surgical RCTs, the magnitude of placebo response and how it is affected by duration of follow-up.

**Methods:**

The databases of MEDLINE, EMBASE, the Cochrane Central Register of Controlled Trials and ClinicalTrials.gov were searched from their inception to 20 October 2015 for studies comparing the efficacy of a surgical intervention with placebo. Inclusion was not limited to any particular condition, intervention, outcome or patient population. The magnitude of placebo response was estimated using standardized mean differences (SMDs). Study estimates were pooled using random effects meta-analysis. Potential sources of heterogeneity were evaluated using stratification and meta-regression.

**Results:**

Database searches returned 88 studies, but for 41 studies SMDs could not be calculated, leaving 47 trials (involving 1744 participants) eligible for inclusion. There were no temporal changes in placebo response within the analysed trials. Meta-regression analysis showed that duration of follow-up did not have a significant effect on the magnitude of the placebo response and that the strongest predictor of placebo response was subjectivity of the outcome. The pooled effect in the placebo arm of studies with subjective outcomes was large (0.64, 95% CI 0.5 to 0.8) and remained significantly different from zero regardless of the duration of follow-up, whereas for objective outcomes, the effect was small (0.11, 95% CI 0.04 to 0.26) or non-significant across all time points.

**Conclusions:**

This is the first study to investigate the temporal changes of placebo response in surgical trials and the first to investigate the sources of heterogeneity of placebo response. Placebo response in surgical trials was large for subjective outcomes, persisting as a time-invariant effect throughout blinded follow-up. Therefore, placebo response cannot be minimized in these types of outcomes through their appraisal at alternative time points. The analyses suggest that objective outcomes may be preferable as trial end-points. Where subjective outcomes are of primary interest, a placebo arm is necessary to control for placebo response.

**Electronic supplementary material:**

The online version of this article (doi:10.1186/s13063-016-1720-7) contains supplementary material, which is available to authorized users.

## Background

There is an increasing interest in surgical randomized controlled trials (RCTs) with a placebo arm [1]. However, the magnitude and duration of the placebo response following surgical procedures, i.e. the effect not related to the main surgical maneuvers, has not been characterized. Some authors have suggested that the placebo response in surgery is large [2] and, like pharmacological treatment, has a time-effect curve, with a peak and a carry-over effect [3].

Studies of non-surgical placebos have demonstrated that the outcome type affects the magnitude of placebo response, with subjective outcomes resulting in a larger placebo response than objective ones [4]. Moreover, in studies with subjective outcomes, placebo response was larger in pain outcomes than in function outcomes [5]. Other trial characteristics that have been suggested to affect placebo response include the number of subjects [4, 6], the frequency of face-to-face visits [7] or placebo administration [6], the randomization ratio [7], subject baseline pain intensity [7], the study design (parallel versus cross-over) and the location of the study (Europe versus North America) [8].

Two recent reviews of surgical RCTs have observed a large effect in the placebo arm which explained about 80% of the variance of the effect within the surgical arm [9] and accounted for 65% of the overall improvement [10]. However, the dependence of placebo response on the time of the follow-up assessment has not been investigated. Our previous review [1] focussed on comparing the active and placebo arms of surgical RCTs in terms of “harms” (assessed as serious adverse events) and benefits (estimated as the effect size in the surgical arm in comparison to the placebo arm) [1]. Within the included studies, the benefits of the surgical intervention relative to the placebo were generally small, and we did not specifically investigate improvement within the placebo arm.

**Fig. 1:**
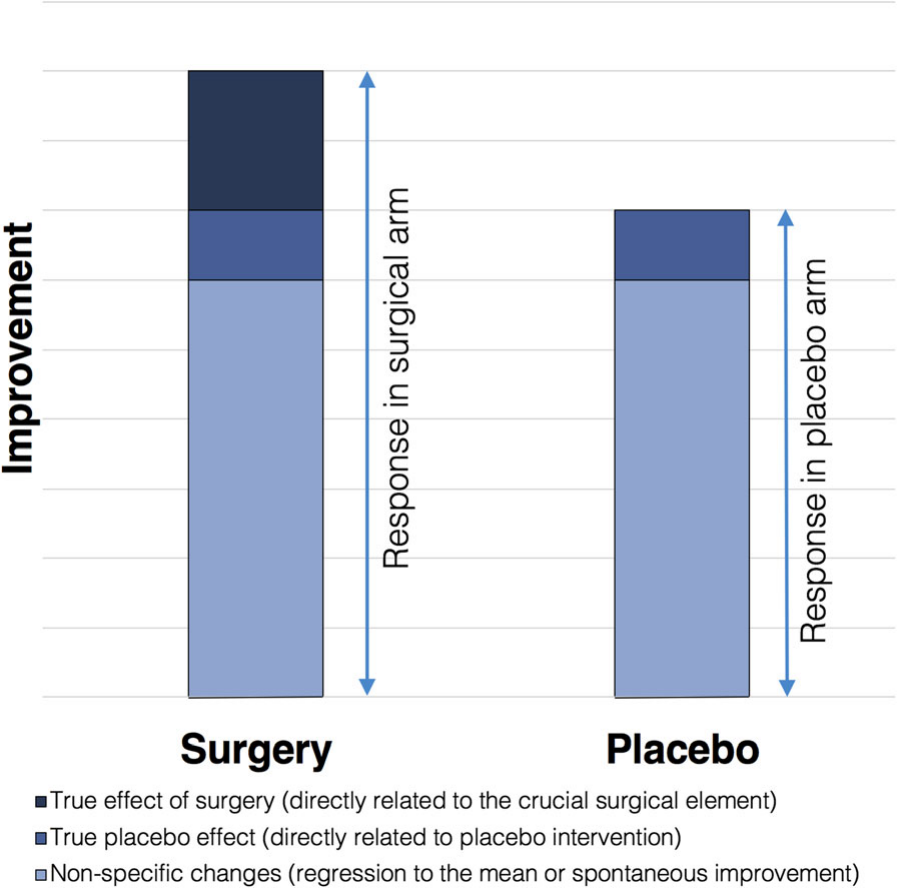
Elements contributing to improvement in the surgical and placebo arms

The terms “placebo effect” and “placebo response” are often used interchangeably [6, 7]. In this paper, the term “placebo effect” was used to refer to the “true placebo effect”, i.e. the changes in response associated with the meaning of treatment [11], conditioning and expectations [12]. The term “placebo response” was used to describe the improvement, i.e. the difference between the baseline and follow-up measures, in the placebo arm (Fig. 1). Differences between placebo effect and placebo response have been discussed in depth previously by Ernst and Resch [12]. The magnitude of placebo response represents a compound effect, only a portion of which may be attributable to the true placebo effect. The remainder of the response within the placebo arm may reflect non-specific effects. These include statistical phenomena (such as regression to mean), biological aspects of disease progression or natural history of the disease and psychological effects of being observed by and receiving attention from clinical staff.

It is assumed that surgery has a large effect on study outcomes and therefore the difference between the surgical and placebo arm is large [13]. However, reviews of published placebo-controlled surgical trials have demonstrated that the effect size in both arms can be substantial, whilst the difference in effect between the arms is often small [1, 9]. This suggests that some surgical procedures may be truly ineffective, with most of the observed improvement attributable to the effect of non-specific factors. Alternatively, some trials may fail to demonstrate the superiority of treatment over placebo, not because of the ineffectiveness of the procedure, but because the observed effect of surgery versus placebo is small [1, 7]. In the latter scenario, a larger sample size would be required to detect a difference between the groups.

Understanding the characteristics of response in the placebo arm is important for both the design and interpretation of placebo-controlled RCTs. If improvement in the placebo arm changes over time, the choice of assessment timing may affect the results of the trial.

The aim of this study was to estimate the magnitude of the placebo response in surgical RCTs and examine the factors on which it depends: mainly whether the effect changes over time and for how long it persists.

## Methods

### Search strategy

The criteria used to identify placebo-controlled surgical RCTs have been described previously [1]. In brief, the databases of Medical Literature Analysis and Retrieval System Online (MEDLINE), Excerpta Medica dataBASE (EMBASE) and the Cochrane Central Register of Controlled Trials were searched for RCTs in which the efficacy of surgery was compared to a surgical placebo. (Details of the search terms can be found in Additional file 1.) ClinicalTrials.gov, a database of registered RCTs, was also queried to identify any recently completed studies with published results. The searches were performed on 20 October 2015.

Five reviewers (KAW, IR, BJFD, JAS, BGF) screened the initial set of abstracts identified from the database search. The reviewers independently assessed the eligibility of each study abstract and the full text, and the final list of included studies was agreed upon by consensus.

### Eligibility criteria

Studies were eligible for inclusion if they investigated the efficacy of surgery through comparison to a placebo procedure, and if they reported a continuous primary outcome measure for which the effect size could be calculated. Trials were also included if they specified a non-continuous primary outcome, i.e. a dichotomization of a continuous measure, but provided the mean and standard deviation (SD) for the measure on which the primary outcome was based. For example, trials were included in which the outcome was defined as a 50% improvement in pain but the mean and SD or 95% confidence intervals (CIs) for the pain scores were reported.

“Surgery” was defined as any interventional procedure that changed the anatomy and required a skin incision or the use of endoscopic techniques. Dental studies and invasive procedures used to deliver a pharmacological substance or stem cells, or that aimed to alleviate symptoms by modulation, stimulation or denervation were excluded.

The term “placebo” was used to refer to a surgical placebo, a sham surgery or an imitation procedure intended to mimic the active intervention, including the scenario where a scope was inserted but no active procedure was performed whilst patients were sedated or under general anaesthesia and could not determine whether or not they had received the surgical intervention. Trial inclusion was not limited to any particular condition, intervention, outcome or patient population.

### Data extraction

The main characteristics of each trial were entered in the standardized data extraction form, including the publication year, country in which the trial was conducted, blinding (who was blinded), randomization ratio, key characteristics of the surgical and placebo procedure (including concomitant standard treatment such as levodopa in Parkinson’s trials or analgesics in pain trials) as well as outcome details (including the type of outcome and the primary assessment time point). Outcomes were classified as “subjective”, i.e. patient-reported and depending on the patients’ perception and cooperation, “assessed”, i.e. subjective ratings judged by external assessors or “objective”, i.e. measured using devices or laboratory tests and independent of patients’ or observers’ perception, for example weight.

For each study time point of each trial, the following data were recorded: the mean and SD of the outcome in the placebo arm, the number of individuals in the placebo arm, the time point number (1^st^ follow-up, 2^nd^ follow-up, etc.) and time since the placebo procedure was conducted. For trials that only reported the outcomes in figures, values were extracted from the figures. Where the SD of the outcome was not reported at follow-up, the SD of the baseline value was used, under the assumption that there was no strong mean-variance relationship.

Data were extracted for all primary outcomes. If there was more than one primary outcome or the primary outcome was not defined, the outcome used in the sample size calculation was chosen. Where neither of these was reported, the first outcome mentioned in the abstract was used. No attempts were made to contact the authors of identified trials. Where necessary, the direction of effect was reversed, so that improvement was consistently presented in the same direction, i.e. as a reduction in SMD. In cross-over trials, the data from the cross-over time point were used as the primary assessment time point for the primary study outcome. If the follow-up was longer than the blinding, the data from the last blinded follow-up visit were used.

### Data synthesis and analysis

Study effect size was quantified as standardized mean differences (SMDs), which were calculated from the baseline and follow-up values of the mean and SD of the study outcomes using Cohen’s *d* method [14, 15] at each follow-up time point. A pretest-posttest correlation coefficient (*r*) of 0.5 was used to calculate the standard error of the SMD, if not otherwise reported. The value for *r* was estimated from 11 trials that reported both the SD of the mean and the SD of difference between the means [14]. The median value of *r* in these studies was 0.5, ranging from 0.2 to 0.6. The effects of potentially misspecifying the value of *r* were evaluated in sensitivity analyses.

SMDs greater than 0.8 are usually considered to be large, and SMDs between 0.5 and 0.8 are considered to be moderate [15, 16]; however, there is no consensus on the interpretation of the magnitude of effect sizes.

To estimate the magnitude of placebo response, a meta-analysis was used to calculate the pooled effect in the placebo arms across all the trials with continuous outcomes, subgrouped by outcome type, as in the meta-analysis by Hróbjartsson and Gøtzsche [17]. The magnitude of the placebo response was calculated as the effect size for the primary outcome at the primary assessment time point.

The effect of follow-up time on placebo response was evaluated by meta-regression. Time, in months, was entered as a continuous variable. Only one follow-up visit was used per trial, i.e. the primary assessment time point. Other potential trial-level factors reported in the literature as affecting the magnitude of placebo response (or placebo effect) were also investigated, including type of outcome (subjective versus objective and assessed versus objective), study location (North America, i.e. the USA or Canada, versus other countries, where multicenter trials were classified as “other countries”), blinding (whether assessor was reported as blinded or not; blinding of patients was an inclusion criterion) and randomization ratio (balanced, i.e. 1:1 versus unbalanced, i.e. with a larger number of patients randomized to the surgical arm). Additionally, we analysed whether the presence of a concomitant standard treatment, either throughout follow-up or as a rescue medication, had an effect on the magnitude of response in the placebo arm.

To further investigate temporal changes over the course of the observed follow-up, meta-analyses were performed in which pooled SMD estimates were grouped by follow-up time (in months ± 2 weeks).

Random-effects models were used in all statistical analyses to account for the potentially high levels of between-study heterogeneity anticipated from pooling different types of surgical trials, outcomes and patient populations. For standard meta-analysis models, the DerSimonian and Laird method was used to derive between-study heterogeneity estimates, whilst for meta-regression models, restricted maximum likelihood was used. In all instances, between-study heterogeneity was quantified as the *I* ^2^ statistic [18]. In order to estimate the magnitude of placebo response in a future trial, we also calculated 95% prediction intervals [19].

The risk of bias in the included studies was assessed using the risk of bias tool criteria recommended by the Cochrane guidelines [20, 21]. Funnel plots [22] and Egger’s test [23] were used to determine the presence of possible publication bias.

The statistical analysis was performed in Stata 12.1 SE [24] using the “metan” [25], “metareg” [26], “metabias” [27] and “metafunnel” [28] packages.

## Results

### Study selection

**Fig. 2:**
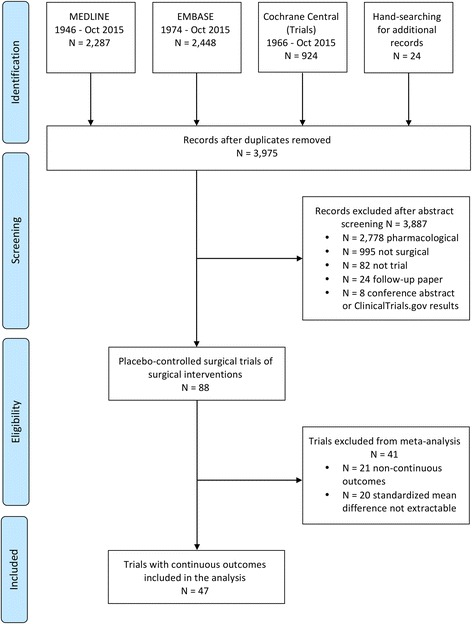
PRISMA flow diagram

The search identified 88 full-text papers reporting surgical RCTs with a placebo arm. Forty-one studies were excluded because either they reported non-continuous primary outcomes (*n* = 21/47, 45%) or they reported a median value or a change score, from which the SMD could not be calculated (*n* = 20/47, 43%). This left 47 trials (involving 1744 participants) eligible for inclusion in the analysis. See the Preferred Reporting Items for Systematic Reviews and Meta-Analyses (PRISMA) flow diagram [29] in Fig. 2, the PRISMA checklist in Additional file 2 and the list of all identified placebo-controlled RCTs in Additional file 3.

### Study characteristics

The median number of patients randomized to the placebo arm was 26, inter-quartile range (IQR) 16–55. The included trials investigated heterogeneous conditions and procedures. The characteristics of the included trials, such as investigated condition, type of surgery, country in which the trial was undertaken, type of blinding, outcomes and randomization ratio are presented in Additional file 4. Nearly two-thirds of the trials used a subjective primary outcome (*n* = 31/47, 66%). In six trials (13%), including three studies on Parkinson’s disease, the outcome was rated by a blinded assessor, and in ten trials (21%) the outcome was objective.

All but seven trials (15%) were double-blinded (blinded patients and assessors). Two of the single-blinded (only patients blinded) trials had an objective outcome (weight loss or glycated hemoglobin levels), whilst the other five used patient-reported outcomes (quality of life or severity of symptoms).

The randomization ratio was balanced, i.e. 1:1, in 32 trials (68%) and unbalanced in favour of the active treatment in 14 trials (2:1 in 11 trials and 3:1 in 3 trials). One study also included an observational group and had a randomization ratio of 1:1:1. This study was classified as having balanced randomization because the observational group was unblinded; therefore, the randomization of the blinded patients was balanced between the surgery and placebo groups.

A cross-over design was used in 3 studies, in 22 studies patients were given an option to cross over after the unblinding, whilst 22 trials did not report whether patients were given the option to cross over.

In 40 trials, there was only one treatment visit, 3 offered “retreatment” and 4 used devices for a prolonged period of time.

Apart from the anaesthesia or analgesia necessary for interventional procedures, most of the trials (*n* = 37/47, 79%) also offered all patients a standard pharmacological treatment, rescue medication or other treatment such as diet or exercise. Only ten trials (21%) did not report using any additional treatment in the placebo arm.

The median primary assessment time point was 6 months, ranging from 1 day to 2 years. In half of the trials, the primary outcome was assessed at the first follow-up time point (*n* = 25/47, 53%). The largest number of follow-up time points between the procedure and the primary outcome assessment was nine. About two-thirds of studies used a subjective primary outcome (*n* = 30/47, 64%), in seven trials the outcome was rated by a blinded assessor and in ten trials the outcome was objective.

### Placebo response across all trials

**Fig. 3:**
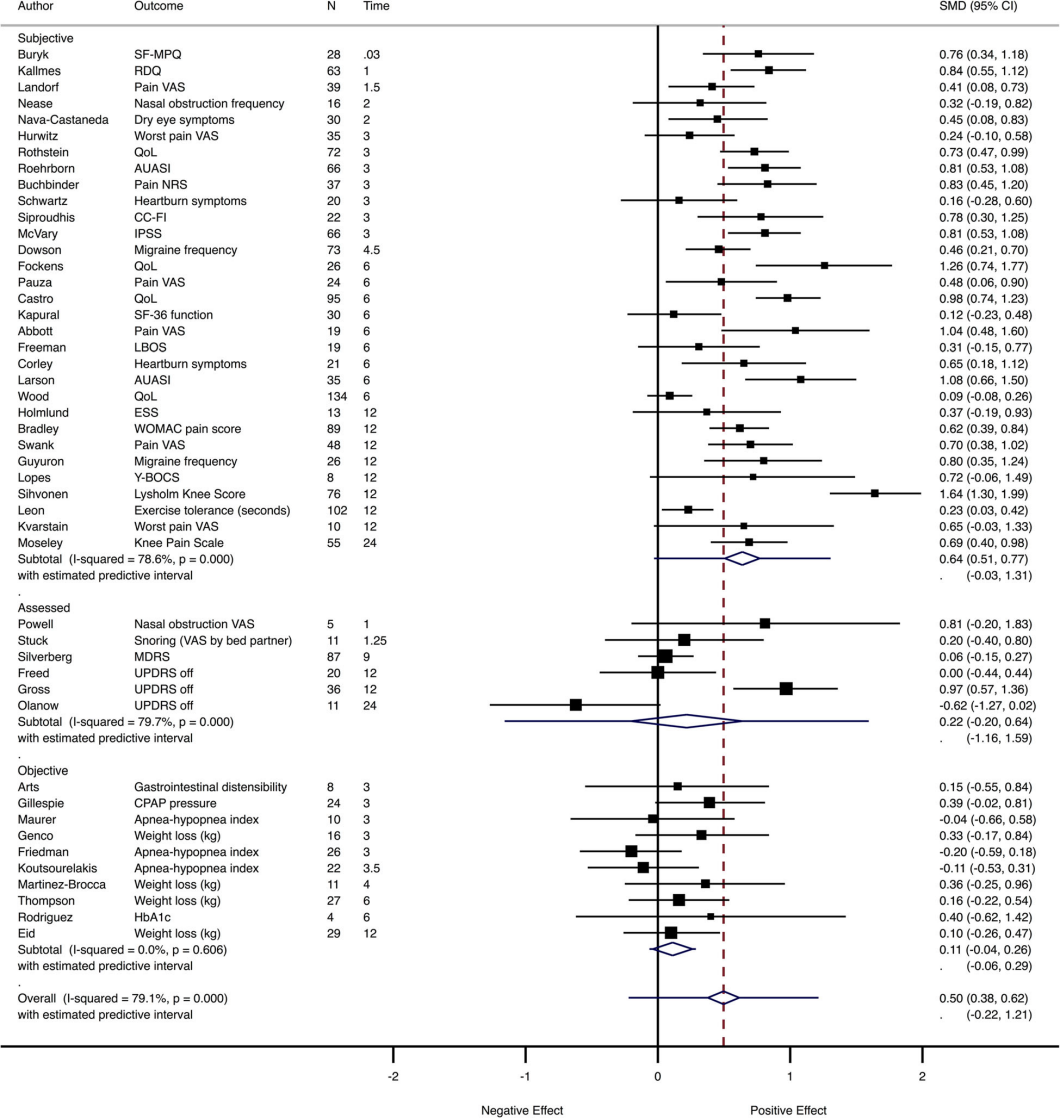
Meta-analysis of all trials. Pooled SMD estimates across all trials assessed at the primary assessment time point, grouped by outcome type and ordered by duration of follow-up (in months). *AUASI* American Urological Association Symptom Index, *BMI* body mass index, *CC-FI* Cleveland Clinic Florida - Faecal Incontinence score, *CPAP* continuous positive airway pressure, *ESS* Epworth Sleepiness Scale, *HbA1c* glycated hemoglobin, *IBFAT* Infant Breastfeeding Assessment Tool, *IPSS* International Prostate Symptom Score, *LBOS* Low Back Outcome Score, *MDRS* Mattis Dementia Rating Scale, *NRS* numerical rating scale, *QoL* quality of life, *RDQ* Roland–Morris Disability Questionnaire, *SF-36* Short Form Health Status Questionnaire, *SF-MPQ* McGill Pain Questionnaire, *UPDRS* Unified Parkinson’s Disease Rating Scale, *VAS* visual analogue scale, *WOMAC* Western Ontario and McMaster Universities Osteoarthritis Index, *Y-BOCS* Yale–Brown Obsessive Compulsive Scale

The pooled estimate for placebo response across all trials was 0.50 (95% CI 0.38–0.62) with the 95% prediction interval for a future trial (−0.22 to 1.21). Heterogeneity was substantial (*I* ^2^ = 79%). When stratified by outcome type, the effect size was moderate for subjective outcomes (0.64, 95% CI 0.51–0.77), but there was no significant pooled effect for assessed (0.22, 95% CI −0.20 to 0.64) or objective outcomes (0.11, 95% CI −0.04 to 0.26) (Fig. 3). For trials with assessed outcomes, the pooled effect was affected by the trial on advanced Parkinson’s disease [30], in which the main outcome assessment was after 2 years, during which time patients deteriorated as a result of disease progression. Within each outcome type, trials were ordered by follow-up time, but there was no clear effect of duration of follow-up on the magnitude of the SMD (Fig. 3).

### Analysis of sources of heterogeneity in all trials

**Fig. 4:**
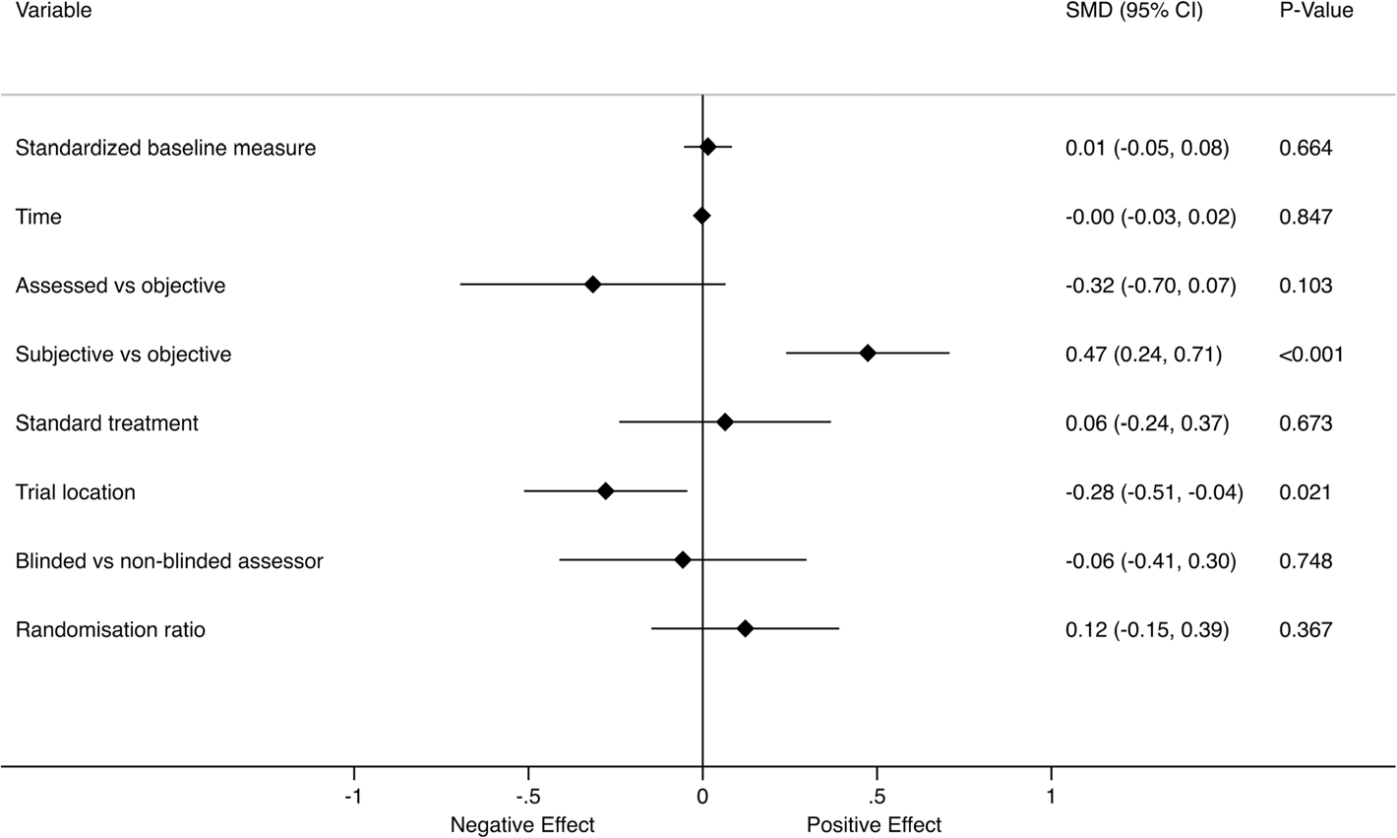
Univariable meta-regression of the factors affecting the SMD at the primary assessment time point

**Fig. 5:**
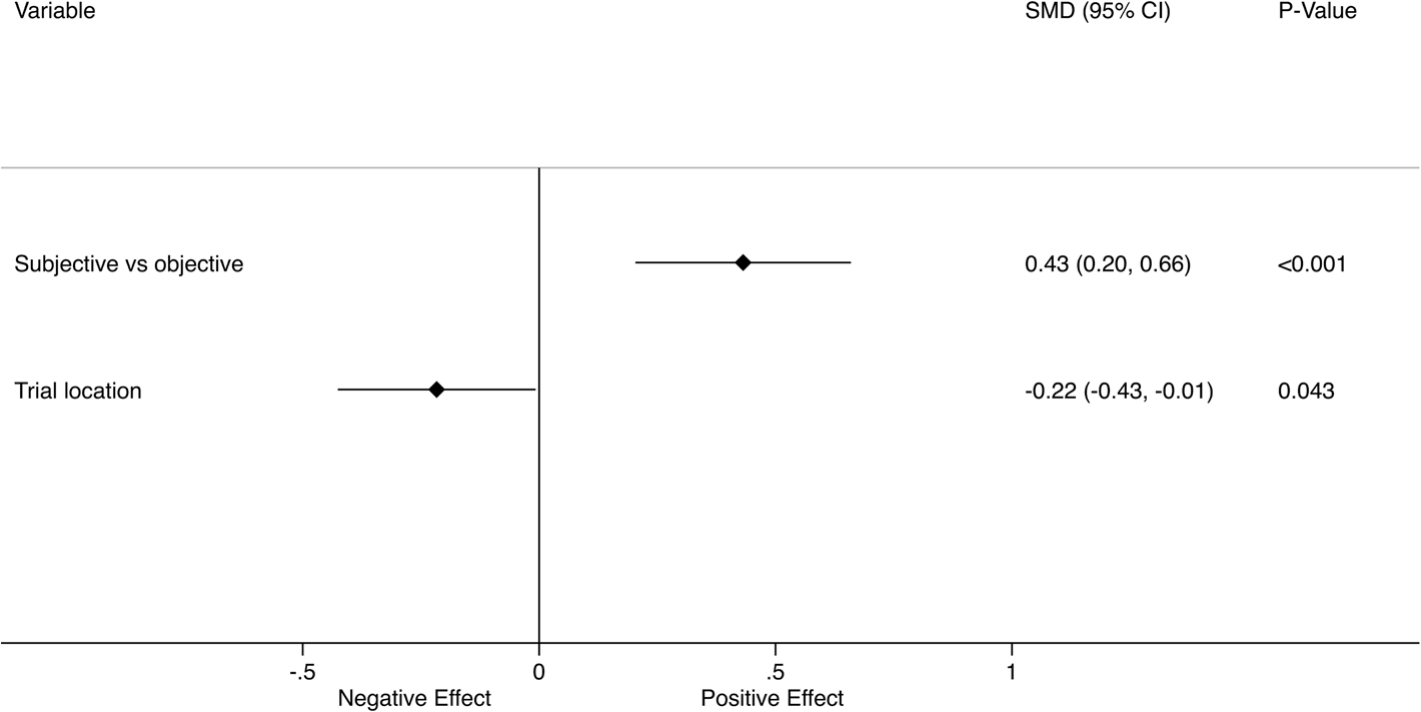
Multivariable meta-regression of the factors affecting the SMD at the primary assessment time point

Duration of follow-up did not have a significant effect on placebo response in univariable meta-regression analyses (Fig. 4). The randomization ratio, the use of concomitant standard treatment and the evaluation of assessed outcomes also had no statistically significant effect on placebo response in univariable meta-regression analyses. The only factors significantly associated with the magnitude of response were the subjectivity of outcome and trial location. When these two variables were combined as predictors in a multivariable meta-regression analysis, both remained significant (Fig. 5).

### Analysis of temporal changes in trials with similar outcomes

**Fig. 6:**
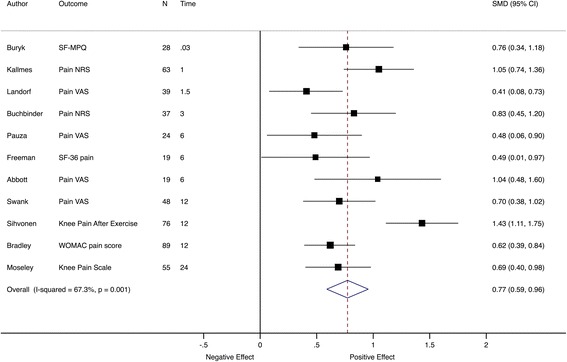
Meta-analysis of pain trials at the primary assessment time point. Pooled SMD estimates for 11 trials with pain intensity as the primary outcome at the primary assessment time point, ordered by follow-up time (in months). *SF-MPQ* McGill Pain Questionnaire, *NRS* numerical rating scale, *VAS* visual analogue scale, *SF-36* Short Form Health Status Questionnaire, *WOMA*C Western Ontario and McMaster Universities Osteoarthritis Index

Analyses were limited to trials with similar outcomes, in order to make the trials more comparable and reduce between-trial heterogeneity. The most common subjective primary outcome was average pain intensity (*n* = 11/47, 23%). For these trials, the pooled effect size was moderate to large, 0.77 (95% CI 0.59–0.96) (Fig. 6).

**Fig. 7:**
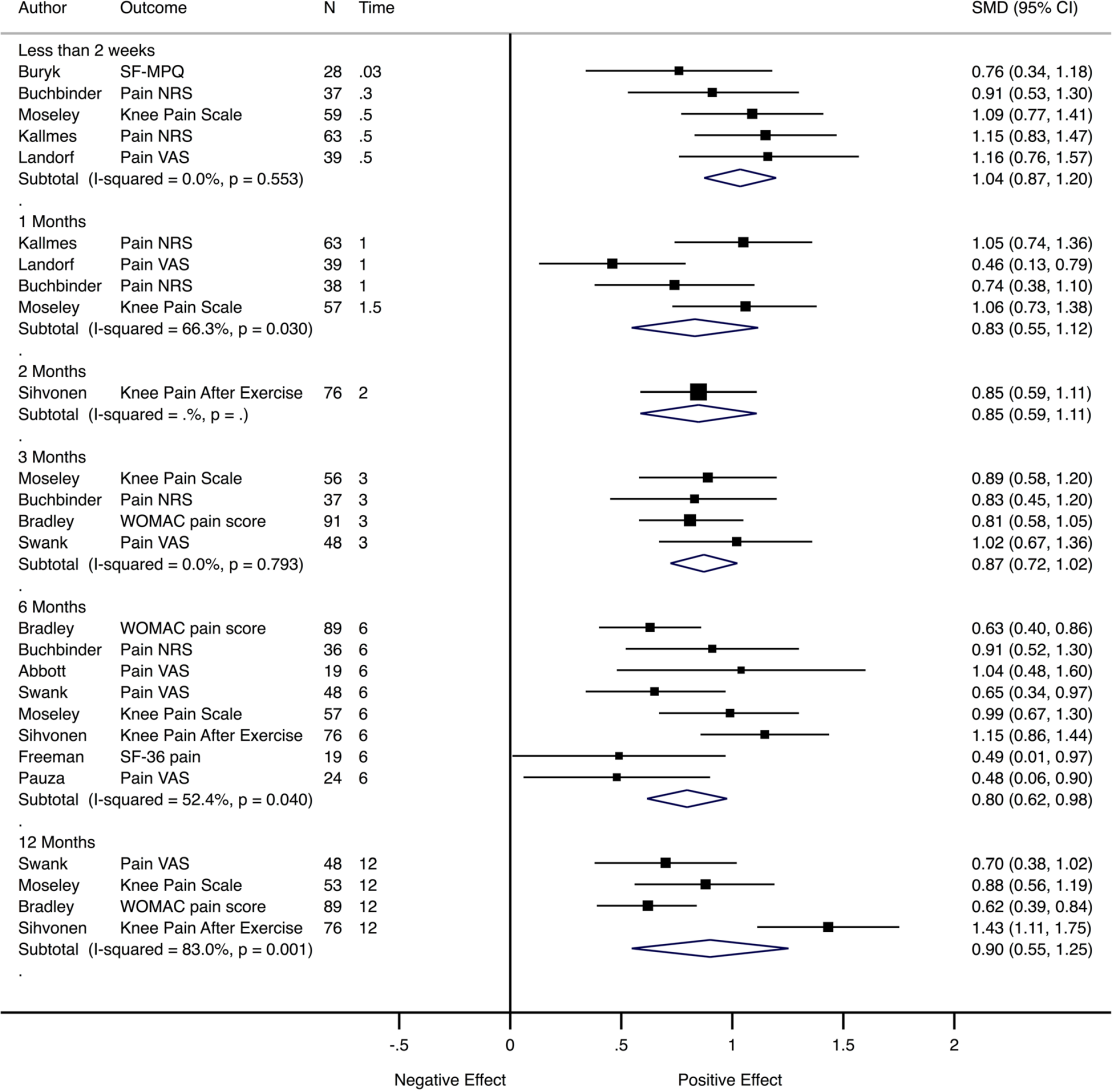
Meta-analysis of pain trials grouped by follow-up time. Pooled SMD estimates for 11 trials with pain intensity as the primary outcome at multiple follow-up visits, grouped by follow-up time (in months). *SF-MPQ* McGill Pain Questionnaire, *NRS* numerical rating scale, *VAS* visual analogue scale, *SF-36* Short Form Health Status Questionnaire, *WOMAC* Western Ontario and McMaster Universities Osteoarthritis Index

When pain intensity effect sizes were grouped by time to follow-up, the pooled effect sizes were large and comparable across time points, from 1.04 (95% CI 0.87–to 1.2) at 2 weeks to 0.80 (95% CI 0.62–to 0.98) at 6 months and 0.90 (95% CI 0.55–to 1.25) at 12 months (Fig. 7).

**Fig. 8:**
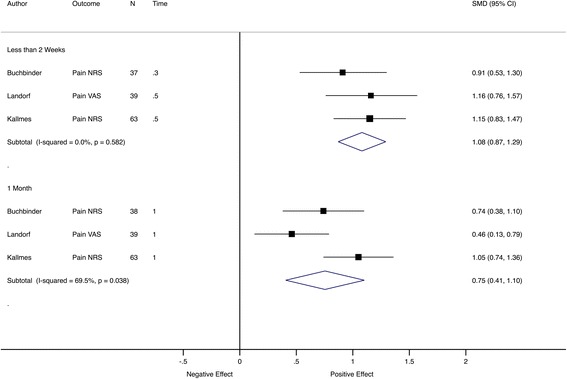
Meta-analysis of pain trials with comparable early follow-up times. Pooled SMD estimates for three trials with pain intensity as the primary outcome and follow-up visits before 2 weeks and at 1 month, ordered by sample size within subgroup. *NRS* numerical rating scale, *VAS* visual analogue scale

**Fig. 9:**
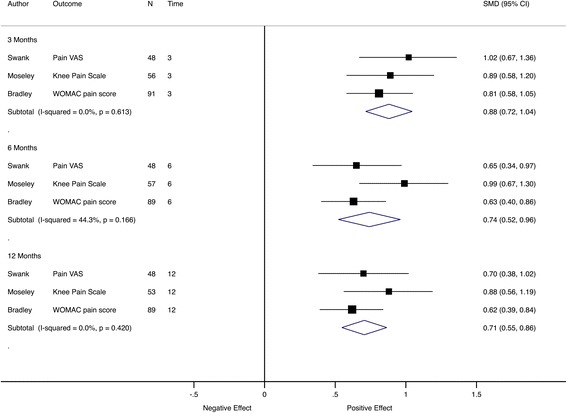
Meta-analysis of pain trials with comparable late follow-up visits. Pooled SMD estimates for three trials with pain intensity as the primary outcome and follow-up visits at 3, 6 and 12 months, ordered by sample size within subgroup. *NRS* numerical rating scale, *VAS* visual analogue scale, *WOMAC* Western Ontario and McMaster Universities Osteoarthritis Index

In Fig. 7, different trials contributed to the pooled effect in each subgroup; therefore, analysis was performed using only trials with the same outcomes and similar follow-up timings. For most time points, only a relatively small number of studies contributed data to the pooled estimate. There were three trials [31–33] with pain as an outcome and assessment at 2 weeks and 1 month (Fig. 8) and another three trials [34–36] with pain as the outcome and assessments at 3, 6 and 12 months (Fig. 9). This analysis showed that the effect size was comparable between follow-up time points.

**Fig. 10:**
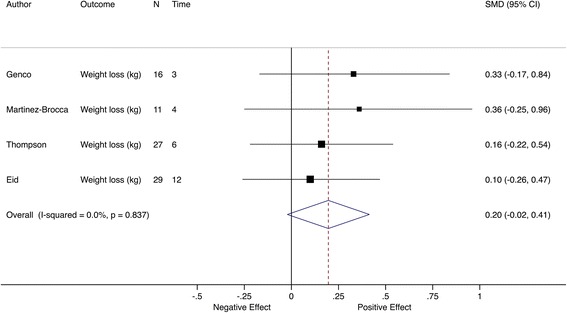
Meta-analysis of weight loss trials at the primary assessment time point. Pooled SMD estimates for four trials with weight loss as the primary outcome, ordered by follow-up time (in months)

**Fig. 11:**
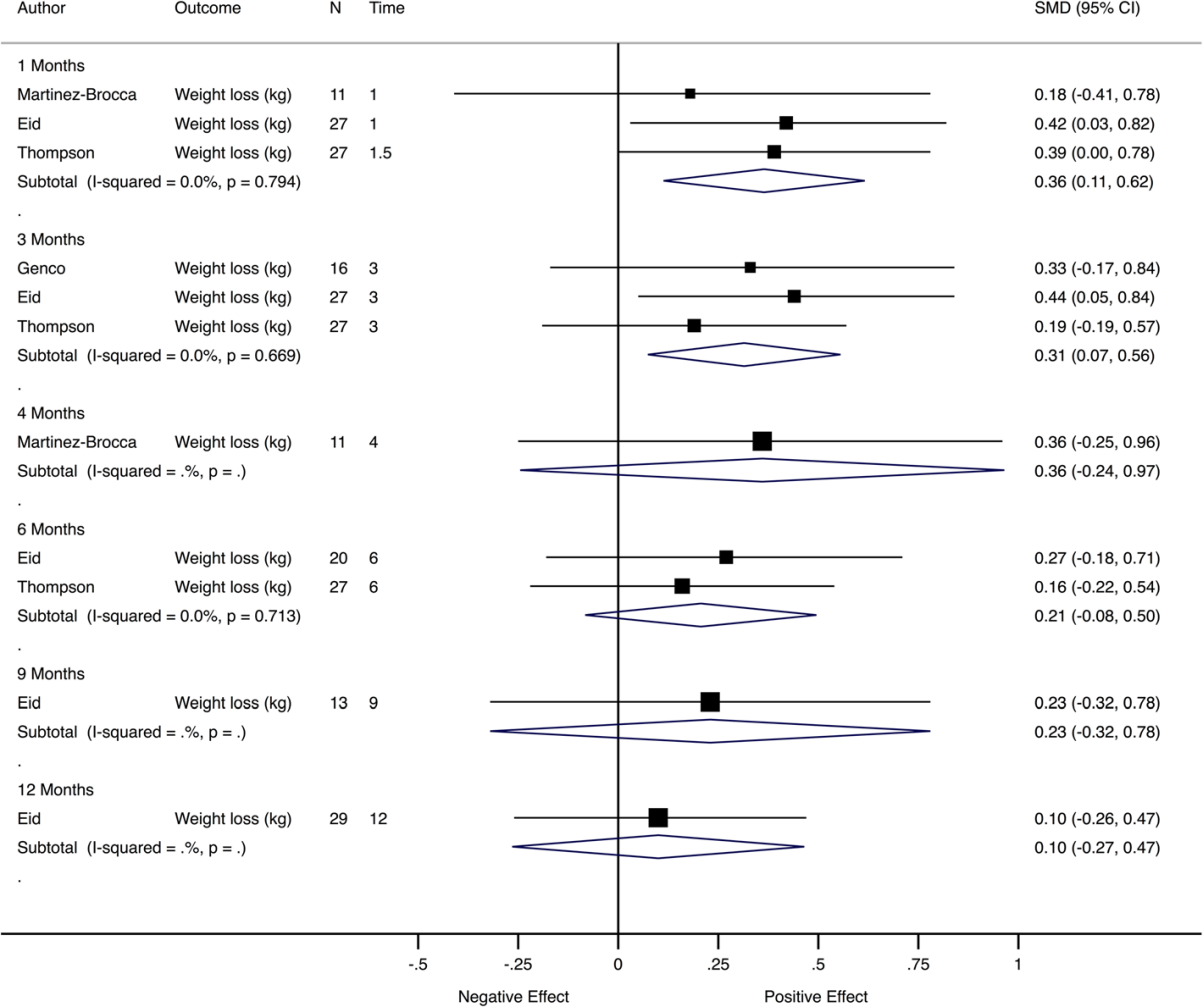
Meta-analysis of weight loss trials grouped by follow-up time. Pooled SMD estimates for four trials with weight loss as the primary outcome, grouped by follow-up time (in months)

The most common objective outcome was weight loss, constituting the primary outcome in four trials (*n* = 4/47, 9%). For these trials, there was a smaller estimated pooled effect with no clear statistical evidence of a difference, SMD = 0.20 (95% CI −0.02 to 0.41; *I* ^2^ = 0) (Fig. 10). A significant pooled effect at the first two follow-up time points was driven by the trial by Eid et al. [37]. The loss of this significance in subsequent visits could be attributable to diminishing patient adherence to diet and exercise regime with time (Fig. 11).

### Risk of bias assessment

The risk of bias assessment of each trial is given in Additional file 5. The risk of bias was generally low. However, some aspects could not be assessed due to limited reporting. For example, only 57% of the trials clearly described sequence generation and allocation concealment. The blinding procedure was described in enough detail to rule out bias in 79% of trials (*n* = 37/47). In all studies, patients were blinded and in 85% (*n* = 40/47) assessors were also blinded. All trials reported the primary outcomes, but only 57% (*n* = 27/47) used a single primary outcome. Thirty-two percent of the studies (*n* = 15/47) did not report results of the intention-to-treat analysis.

**Fig. 12:**
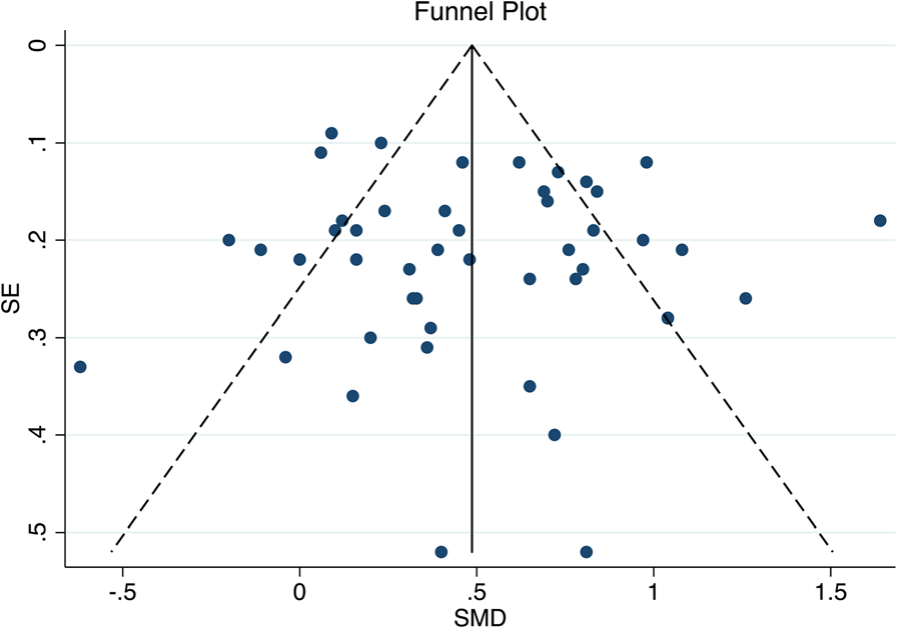
Funnel plot to determine the presence of publication bias. Plot of standard error (*SE*) of SMD versus SMD

The funnel plot in Fig. 12 appeared symmetrical, implying an absence of publication bias. This observation was corroborated by the results of Egger’s test, which was unable to determine the presence of statistically significant plot asymmetry (*p* = 1.00). Several studies fell outside the 95% CIs of the funnel plot in Fig. 12, with a large number of these having relatively high precision in their effect size estimates. This may have resulted from pooling of trials that were largely heterogeneous. A similar distribution of study effect sizes has been reported in a Cochrane review of placebo interventions [38].

### Sensitivity analysis

The meta-analysis model of the primary analysis of all 47 trials was re-run using *r* values of 0.3 and 0.7. The pooled effect across all trials was 0.50 (CI 0.38–0.62, *I* ^2^ = 67%) for *r* = 0.2 and 0.50 (CI 0.38–to 0.61, *I* ^2^ = 83%) for *r* = 0.6. This resulted in minimal differences in the pooled effects for each type of outcome.

## Discussion

### Main findings

There were no temporal changes in placebo response within the analysed trials, and the response remained comparable between follow-up time points. Meta-regression analysis showed that the duration of follow-up had no effect on placebo response. The magnitude of response depended primarily on the outcome type, i.e. whether the outcome was subjective or objective. For trials with subjective outcomes, the pooled effect size of placebo response was large and it persisted for the duration of the blinded follow-up; i.e. the follow-up values did not return to baseline. However, for trials with objective outcomes, the effect was small or not significant across all time points.

### Strengths and limitations

This study is a comprehensive search and analysis of placebo-controlled surgical RCTs identified through a systematic search. It is, to the best of our knowledge, the first study to characterize temporal changes in response in the placebo arm of surgical RCTs.

Data could not be included from 41 out of 88 identified trials, because the primary outcomes were binary, reported as medians with IQRs or were not associated with any data that could be extracted from the text or the figures. We did not include secondary outcomes.

Within studies eligible for inclusion, analyses were limited to the use of aggregate data, as reported by trial authors, rather than individual patient data. The lack of precision of our analyses reflected the relatively low number and small size of included studies and therefore did not investigate the effect of patient-level factors such as age, sex or expectations, which have been suggested to affect the magnitude of placebo response.

This study did not assess the effect or success of blinding, as these checks are unable to distinguish between the ineffectiveness of blinding from patient “hunches” regarding perceived treatment efficacy [39]. The effect of re-treatment could also not be investigated, as most reviewed studies used a “one-off” intervention [1], with only three studies using repeated placebo procedures. The number of follow-up time points, i.e. whether the primary outcome was assessed at the first or at a subsequent visit, was not used in the meta-regression because it was correlated with follow-up time.

We have performed a meta-analysis of placebo response under the assumption that the placebo intervention is inactive, and therefore comparable across trials. Few trials evaluated similar conditions or interventions or used comparable follow-up time points; for example, the included 47 trials investigated 28 different conditions. Thus, our ability to explain the potential sources of heterogeneity through subgroup and meta-regression analysis was limited, and much of the heterogeneity remained unexplained. Some heterogeneity may have been related to factors we did not investigate. It has been demonstrated by Vase and colleagues [7] that most of the between-trial heterogeneity is caused by patient-level characteristics (to which we had no access) rather than trial-level factors.

### Interpretation

It is likely that the placebo response is not just the true placebo effect, which has been demonstrated to be small [4, 38], but possibly also the result of concomitant treatment, natural history of the disease and regression to the mean, which are discussed in more detail in the following paragraphs.

We could not investigate the extent to which placebo response is caused by the true placebo effect, because only one reviewed trial included a non-interventional group [40] that could be used to control for the natural history of disease and other non-specific changes [4]. It is plausible that the sustained large placebo response in trials with subjective outcomes may be a result of the physiological effects directly related to placebo [9]. From the extensive analysis by Hróbjartsson and Gøtzsche [4, 17, 41, 42], it is thought that the magnitude of the true placebo effect is generally small in clinical trials, and that placebo response is primarily driven by non-specific bias. A recent meta-analysis of pharmacological trials showed that the effect size in the placebo arm was indeed larger than in the non-interventional control arm, which suggests that placebo manipulation exerts some additional effect beyond non-specific changes [6].

The persistence of placebo response has been explained in terms of “the learning theory”; i.e. after the withdrawal of a cue the response does not stop immediately but gradually declines [43]. However, in the analysed trials there was no significant reduction of the placebo response.

Some of the improvement in the placebo arm might be related to the effect of concomitant treatment. It is often assumed that the placebo intervention is truly inactive and that it does not affect the response in the placebo group by means other than psychological [44]. However, most of the trials in this review used concomitant treatments liable to induce physiological or pharmacological effects, i.e. standard treatments such as anti-parkinsonian drugs, rescue medications such as analgesics or lifestyle modifications, for example diet or exercise. Therefore, although this study aimed to include only purely surgical trials, the response in the placebo arm might encompass other treatments as opposed to being solely the true placebo effect and bias. This may explain a larger effect in trials on obesity at the beginning of the follow-up period with diet and exercise having short-term effects but not long-term effects.

Part of the response in the placebo arm may be associated with non-specific factors, such as regression to the mean and the natural history of the disease. For example, a meta-analysis of pain trials reported that higher pain scores at baseline correlated with a larger placebo response [7]. It is likely that the sustained positive change in the placebo arm of trials with subjective outcomes, especially pain, is to some degree an effect of “being in the trial”, i.e. receiving additional attention and support from the clinical staff; the latter being reported as the most powerful of the non-specific effects [11]. However, it is also likely that some of the observed change may be the result of patients reporting improvement out of politeness [45] or because their ratings change with improved well-being or reduced stress [46].

There have been two recent reviews of placebo effect in surgical trials with a placebo arm. Both studies concentrated primarily on the differences in response between the surgical and placebo arm, and neither of them included a comprehensive analysis of temporal changes in the magnitude of placebo response. Moreover, they differed slightly in definition of surgery and in their eligibility criteria. In one, the authors did not exclude trials investigating an invasive delivery of pharmacologically active substances, for example the intra-articular injection of steroids [9]. In the present study, such trials were not included and analyses were restricted to purely surgical studies, because of possible differences in placebo response (including information provided to the patients and patients’ expectations when a drug treatment was involved) [47]. Another recent review [10] included procedures which were excluded from this study, as they did not fulfil our definition of surgery. Trials with binary outcomes were also excluded, as this analysis was concerned only with changes in the placebo arm, unlike the study by Holtedahl et al. [9] which investigated differences between the surgical and the placebo arm. Cross-over trials and trials with only graphical representations of the outcome data were included. Therefore, only 11 out of 21 studies analysed by Holtedahl et al. [9] and 24 out of 39 trials by Jonas et al. [10] were included in this analysis.

Heterogeneity in this study was higher than in another meta-analysis of surgical RCTs (*I* ^2^ = 76%) [10] but lower than in the meta-analysis of individual patient data from studies on pain in osteoarthritis (*I* ^2^ = 99%) [7]. The high heterogeneity could have been caused by the lack of restrictions on the types of studies eligible for inclusion. However, there is evidence that the condition [7] and treatment procedure may have a weak effect on placebo response [9].

The effect size in the placebo arm was large for subjective outcomes, which is in line with the findings of other reviews [4, 6, 9]. The effect on pain was larger than in a meta-analysis of various therapies on musculoskeletal pain [6], but this may be related to the invasiveness of surgery relative to other treatments [6, 48].

In this analysis, there was no statistically significant effect for assessed outcomes. It is likely that assessed outcomes are less influenced by a placebo effect or bias than subjective ones, but the finding of no statistically significant effect was unexpected, especially as a significant effect for assessed outcomes has been reported elsewhere [41]. The number of included trials was small and the heterogeneity was large; therefore, there may not have been enough statistical precision to detect a genuine effect.

For trials with objective outcomes, there was no statistical evidence of significant effect in the placebo arm. This is in line with the findings of an earlier review, which reported that placebos had no significant effect on objective outcomes [4]. The lack of placebo response found by this study suggests that the bias or non-specific changes may also be small for objective outcomes.

This is the first study to investigate the temporal changes of placebo response in surgical RCTs. There have been attempts to analyse changes of placebo analgesia with time, but the observation period was in the range of minutes [46, 49], hours [43] or weeks [5]. Within these studies, three investigated single administration of a non-invasive placebo, i.e. a capsule or a jelly [43, 46, 49], and one investigated repeated application of an invasive placebo, i.e. sham acupuncture [5]. All placebo procedures resulted in significant pain relief throughout the observation period. However, only one study performed a formal analysis to investigate the effect of time, establishing it not to be significant [46].

This is the first study to use meta-regression to investigate factors affecting placebo response in surgical trials. The duration of follow-up had no effect in either univariable or multivariable analyses. The strongest predictor of placebo response was the outcome being subjective rather than objective, which is in line with other meta-analyses of true placebo effect [17]. Concomitant treatment appeared not to explain the heterogeneity present within this study. Randomization ratio had no effect on the placebo response in our analysis. Results from other trials provide conflicting evidence, with some showing that randomization ratio in favour of active treatment results in a smaller placebo response [7], whilst others show the opposite [50]. Our analysis showed that trials located in North America had a smaller placebo response than those located elsewhere. A meta-analysis of placebo response in acute migraine also reported geographic differences, with more pain-free patients in studies performed in Europe [8]. This has been interpreted as being related to differences in patients’ expectations between countries [51].

### Implications

Placebo response appears to not change with time and persists for as long as patients remain blinded and participating in the trial. Therefore, it may not be possible to minimize or maximize the magnitude of placebo response by changing the timings of follow-up time points [47, 52].

This study showed that the placebo response in surgical trials with subjective outcomes is substantial. Therefore, patient-oriented outcomes such as pain, function or quality of life may not be reliable, and trials using such outcomes may not be able to estimate the true treatment effect [53]. Where the response in both arms is large and the difference between arms is small (which is the situation in some surgical RCTs [1, 10]), even a small degree of bias may diminish the perceived efficacy of the treatment [10, 54] and may require a larger sample size to demonstrate the superiority of an intervention to placebo [55]. Where possible, objective outcomes should be used to assess the efficacy of surgical trials. Where this is not possible, or where subjective outcomes are of primary interest, placebo control could be necessary to control for bias from non-specific and placebo effects. The use of non-interventional groups may also prove useful where assessors seek to disentangle non-specific and placebo effects from placebo response.

Placebo response forms part of the response in the active arm. Therefore, a larger effect in the active arm may be paralleled by a larger response in the placebo arm [56]. In clinical practice, the placebo effect may be stronger than in an RCT because the uncertainty about treatment allocation inherent to a trial might reduce the placebo effect [57, 58]. Earlier analyses reported that the placebo response explains 80% of the variance in the surgical arm [9] and 65% of the effect in the surgical arm (78% for pain and 71% for obesity) [10]. However, most of the analysed trials used concomitant treatment which might have interfered with the true placebo effect. Therefore, in these instances, the “additive model” may not be valid [47]. This model assumes that the placebo response is non-specific, and therefore, the same in the active and in the placebo arm. The implication is that, although we know that improvement in the surgical arm may not just be an effect of the critical surgical maneuver, we do not know the extent to which placebo effect and bias contribute. Furthermore, we do not have a sufficient number of placebo-controlled surgical RCTs to investigate this.

## Conclusions

To the best of our knowledge, this is the first meta-analysis to investigate temporal changes in effect in the placebo groups of surgical RCTs. This paper found evidence that the magnitude of placebo response is not affected by the duration of the follow-up and that this effect persisted for the duration of blinded assessment. The most important factor impacting the size of the effect in the placebo arm is the subjectivity of the outcome measure. Trials investigating subjective outcomes tended to have large effects in the placebo arm, whilst trials investigating assessed or objective outcomes tended to have no clear effect in the placebo arm.

## Additional files

Additional file 1: Search terms: list of search terms used to systematically search literature. (DOCX 79 kb)
Additional file 2: PRISMA checklist. (DOC 61 kb)
Additional file 3: All identified RCTs: list of all surgical RCTs with a placebo arm identified during the systematic review. (PDF 40 kb)
Additional file 4: Included RCTs: list of all surgical RCTs with a placebo arm included in this analysis. (PDF 46 kb)
Additional file 5: Risk of bias: table summarizing possible list of bias items in each included trial. (PDF 27 kb)

## Abbreviations

AUASI: American Urological Association Symptom Index
BMI: Body mass index
CC-FI: Cleveland Clinic Florida - Faecal Incontinence score
CI: Confidence interval
CPAP: Continuous positive airway pressure
EMBASE: Excerpta Medica dataBASE
ESS: Epworth Sleepiness Scale
HbA1c: Glycated hemoglobin
IBFAT: Infant Breastfeeding Assessment Tool
LBOS: Low Back Outcome Score
MDRS: Mattis Dementia Rating Scale
MEDLINE: Medical Literature Analysis and Retrieval System Online
NRS: Numerical rating scale
PRISMA: Preferred Reporting Items for Systematic Reviews and Meta-Analyses
QoL: Quality of life
RCT: Randomized controlled trial
RDQ: Roland–Morris Disability Questionnaire
SD: Standard deviation
SF-36: Short Form Health Status Questionnaire
SF-MPQ: McGill Pain Questionnaire
SMD: Standardized mean difference
UPDRS: Unified Parkinson’s Disease Rating Scale
VAS: Visual analogue scale
WOMAC: Western Ontario and McMaster Universities Osteoarthritis Index
Y-BOCS: Yale–Brown Obsessive Compulsive Scale
