# Supplementary material for: The magnitude and temporal changes of response in the placebo arm of surgical randomized controlled trials: a systematic review and meta-analysis
Source: Trials. 2016 Dec 12;17:589. doi: 10.1186/s13063-016-1720-7 (PMC5154040; doi:10.1186/s13063-016-1720-7)
Supplement: Additional file 3: — All identified RCTs: list of all surgical RCTs with a placebo arm identified during the systematic review. (PDF 40 kb) [file 13063_2016_1720_MOESM3_ESM.pdf]

	Study	Year	Condition	Procedure	Placebo intervention	Country	Included in meta-analysis
1	Abbott et al.	2004	Endometriosis	Laparoscopy + ablation	Laparoscopy	UK	Continuous
2	Arts et al.	2012	GERD	Endoscopy + RF treatment	Endoscopy + setup but no RF delivery	Belgium	Continuous
3	Bradley et al.	2002	Osteoarthritis	Tidal irrigation	Saline injected sub cut. and leg manipulation	USA	Continuous
4	Buchbinder et al.	2009	Osteoporotic vertebral fracture	Vertebroplasty	Injection of anaesthetic but not cement +cephalosporin	Australia	Continuous
5	Buryk et al.	2011	Ankyloglossia	Frenotomy	Frenotomy	USA	Continuous
6	Castro et al.	2010	Severe asthma	Bronchoscopy + radiofrequency treatment	Bronchoscopy + placebo procedure	USA, Canda, Netherlands, Israel, Belgium	Continuous
7	Corley et al.	2003	GERD	Endoscopy + RF treatment	Endoscopy + setup but no RF delivery	USA	Continuous
8	Dowson et al.	2008	Migraine	Patent foramen ovale closure	Skin incision in the groin + transesophageal US + aspirin and clopidogrel - no heparin	UK	Primary not continuous but ES could be calculated
9	Eid et al.	2014	Obesity	Endoscopy + gastroplication (StomaphyX)	Endoscopy	USA	Primary not continuous but ES could be calculated
10	Fockens et al.	2010	GERD	Endoscopy + Gatekeeper prothesis	Endoscopy + saline instead of prothesis and instead of antibiotics	USA , Netherlands	Continuous
11	Freed et al.	2001	Parkinson's disease	Transplantation of dopaminergic neurons	Incomplete trepanation (dura intact) + PET + MRI + phenytoin - sham-transplantation	USA	Primary not continuous but ES could be calculated
12	Freeman et al.	2005	Chronic discogenic pain	Electrothermal therapy	Catheter inserted but not connected + cephazolin +CT	Australia	Continuous
13	Friedman et al.	2008	Sleep apnea/hypopnea	Palatal implant	Identical implementation device without an implant + a-biotics	USA	Continuous
14	Genco et al.	2006	Obesity	Endoscopy + balloon	Endoscopy but not balloon + diet??	Italy	Continuous
15	Gillespie et al.	2010	Sleep apnea/hypopnea	Palatal implant	Identical implementation device without an implant	USA	Continuous
16	Gross et al.	2011	Parkinson's disease	Cell transplantation	Scalp incisions and partial-thickness burr holes + MRI - the same duration	USA, Germany	Continuous
17	Guyuron et al.	2009	Migraine	"Deactivation" of trigger points	Exposure of muscles and nervs without changing their integrity	USA	Primary not continuous but ES could be calculated
18	Holmlund et al.	2014	Snoring	Radiofrequency treatment	Sham	Sweden	Continuous
19	Hurwitz et al.	2014	Cancer	Sonication	Setup but no energy delivery	USA, Canada, Israel, Italy, Russia	Continuous
20	Kallmes et al.	2009	Osteoporotic vertebral fracture	Vertebroplasty	Simulated (audio, sensory, even smell) vertebroplasty - injection of anaesthetic but not cement	USA, UK, Australia	Continuous
21	Kapural et al.	2013	Chronic discogenic pain	Radiofrequency treatment	No energy delivery	USA	Continuous
22	Koutsourelakis et al.	2008	Sleep apnea/hypopnea	Septoplasty	Simulated resection with manipulation of instruments - the same amount of time	Greece	Primary not continuous but ES could be calculated
23	Kvarstein et al.	2009	Chronic discogenic pain	Radiofrequency treatment	Sham	Norway	Continuous
24	Landorf et al.	2013	Plantar callus	Debridgement	Sham	Australia	Continuous
25	Larson et al.	1998	Prostatic hyperplasia	Thermoablation	Sham	USA	Continuous
26	Leon et al.	2005	Coronary disease	Percutaneous myocardial laser revascularisation	Setup but no laser procedure	USA	Continuous
27	Lopes et al.	2014	Obsessive-compulsive disorder	Capsulotomy	Sham	Brazil	Continuous
28	Martinez-Brocca et al.	2007	Obesity	Endoscopy + balloon	Endoscopy	Spain	Continuous
29	Maurer et al.	2012	Sleep apnea/hypopnea	Palatal implant	Identical implementation device without an implant	Germany	Continuous
30	McVary et al.	2014	Prostatic hyperplasia	Inmplant	Sham	USA, Australia	Continuous
31	Moseley et al.	2002	Osteoarthritis	Arthroscopy + debridement OR lavage	Skin incision without arthroscopy	USA	Continuous
32	Navada-Castaneda et al.	2003	Dry eye	Lacrimal occlusion	No occlusion	Mexico	Continuous
33	Nease et al.	2004	Turbinate hypertrophy	Radiofrequency treatment	Sham	USA	Continuous
34	Olanow et al.	2003	Parkinson's disease	Tissue/cells transplantation	Partial burr holes + a-biotics + cyclosporine + PET	USA	Continuous
35	Pauza et al.	2004	Chronic discogenic pain	Intradiscal electrothermal therapy	Introducing a needle onto the disc (visual and auditory feedback)+discography + CT + prophylactic a-biotics + analgesics + rehabilitation.	USA	Continuous
36	Powell et al.	2001	Turbinate hypertrophy	Radiofrequency treatment	Setup but no energy delivery	USA	Continuous
37	Rodriguez et al.	2009	Diabetes mellitus type 2	Endoscopy + bypass liner	Endoscopy	Chile	Continuous
38	Roehrborn et al.	2013	Prostatic hyperplasia	Cystoscopy + prostatic urethral lift	Cystoscopy + simulated procedure	USA, Australia, Canada	Continuous
39	Rothstein et al.	2007	GERD	Endoscopy + plication	Endoscopy + setup but device not activated	USA, Germany, Belgium	Continuous
40	Schwartz et al.	2007	GERD	Endoscopy + EndoCinch plication	Endoscopy + setup without needle and thread loaded	Netherlands	Continuous
41	Sihvonen et al.	2013	Degenerative meniscus tear	Arthroscopic partial meniscectomy	Arthroscopy and sham	Finland	Continuous
42	Silverberg et al.	2008	Alzheimer's disease	Ventriculoperitoneal shunt	Identical shunt but occluded	USA	Continuous
43	Siproudhis et al.	2007	Faecal incontinence	Elastomer implants	Saline injection	France	Continuous
44	Stuck et al.	2005	Snoring	Radiofrequency treatment	Device was inserted but not activated	Germany	Continuous
45	Swank et al.	2003	Abdominal pain	Laparoscopy + adhesiolysis	Laparoscopy	Netherlands	Continuous
46	Thompson et al.	2013	Obesity	Transoral outlet reduction	Sham	USA	Primary not continuous but ES could be calculated
47	Wood et al.	2014	Emphysema	Bronchoscopy + endobronchial valve	Bronchoscopy	USA	Continuous
48	Baeck et al.	2009	Sleep apnea/hypopnea	Radiofrequency treatment	Applicator insertion but no RF delivery	Finland	ES could not be calculated
49	Bajbouj et al.	2009	Globus sensation	Endoscopy + argon plasma coagulation	Endoscopy + connected applicator but no current	Germany	ES could not be calculated
50	Barber et al.	2012	Urinary stress incontinence	Tension-free vaginal sling	Mini-sling sham treatment	USA	Non-continuous
51	Benjamin et al.	1988	Obesity	Endoscopy and balloon	Endoscopy + balloon immitation + diet	USA	ES could not be calculated
52	Cobb et al.	1959	Coronary disease	Internal mammary artery ligation	Skin incision and exposure of vessels but no ligation.	USA	Non-continuous
53	Cote et al.	2012	Pancreatitis	Sphincterotomy	Sham	USA	Non-continuous
54	Cotton et al.	2014	Sphincter of Oddi dysfunction	Endoscopy + sphincterectomy + ERCP	Endoscopy + ERCP	USA	Primary not continuous and no SD for continuous outcomes
55	Davey et al.	2015	Emphysema	Bronchoscopy + endobronchial valve	Bronchoscopy	UK	ES could not be calculated
56	Davys et al.	2005	Plantar callus	Callus debridement	Simulation using blunt-edged scalpel	UK	ES could not be calculated
57	Deviere et al.	2005	GERD	Endoscopy + a copolymer	Endoscopy without implant + prophylactic a-biotics	Germany, Belgium, Italy	Non-continuous
58	Dimond et al.	1960	Coronary disease	Internal mammary artery ligation	Skin incision and exposure of vessels but no ligation	USA	Non-continuous
59	Dollberg et al.	2006	Ankyloglossia	Frenotomy	Sham	Israel	ES could not be calculated
60	Fleischer et al.	1985	Oesophageal varices	Endoscopy + laser	Endoscopy + setup (laser was turned on, a verbal order was given to activate the laser but not used) + cimetidine or antiacids after endoscopy + vasopressin if bleeding persisted	USA	Non-continuous
61	Freitas et al.	1985	Peptic ulcers	Endoscopy + electrocoagulation	Sham + cimetidine	Portugal	Non-continuous
62	Fullarton et al.	1989	Peptic ulcers	Endoscopy + heater probe	Endoscopy + heater probe activated in the gut lumen + ranitidine	UK	Non-continuous
63	Geenen et al.	1989	Sphincter of Oddi dysfunction	Endoscopy + sphincterectomy + ERCP	Endoscopy + device activated in the lumen of the duodenum + ERCP + manometry + morphine/neostigmine provocation test	USA	Non-continuous
64	Geliebter et al.	1990	Obesity	Endoscopy and balloon	Endoscopy + deflated balloon	USA	ES could not be calculated
65	Hartigan et al.	1994	Oesophageal varices	Endoscopy + sclerotherapy	Endoscopy + placebo solution released to the gut lumen	USA	Non-continuous
66	Hogan et al.	1989	Obesity	Endoscopy and balloon	Endoscopy + sham insertion + diet	USA	ES could not be calculated
67	Jarrell et al.	2005	Endometrisis	Laparoscopy + sharp excision	Laparoscopy + biopsy	Canada	ES could not be calculated
68	Laine et al.	1987	Peptic ulcers	Endoscopy + electtrocoagulation	Endoscopy + probe activated in the lumen of the gut	USA	Non-continuous
69	Lee et al.	2012	Obesity	Endoscopy + balloon	Endoscopy + saline	Singapore	ES could not be calculated
70	Lee et al.	2001	Urinary stress incontinence	Autologous fat injection	Saiine	Canada	Non-continuous
71	Lindor et al.	1987	Obesity	Endoscopy and balloon	Endoscopy + empty introducer tube + diet	USA	ES could not be calculated
72	MacLeod et al.	1983	Peptic ulcers	Endoscopy + laser	Endoscopy + cimetidine	UK	Non-continuous
73	Mathus-Vliegen et al.	1990	Obesity	Endoscopy and balloon	Endoscopy + manipulation without balloon insertion + simulated "click" of device disconnection	Netherlands	ES could not be calculated
74	Meshkinpour et al.	1988	Obesity	Endoscopy and balloon	Endoscopy + empty introducer tube + simulation of inflation process	USA	ES could not be calculated
75	Montgomery et al.	2006	GERD	Endoscopy + EndoCinch plication	Endoscopy - the same duration	Sweden	ES could not be calculated
76	Ponce et al.	2015	Obesity	Endoscopy and balloon	Endoscopy + balloon immitation + diet	USA	ES could not be calculated
77	Salem et al.	2004	Coronary disease	Percutaneous myocardial laser revascularisation	Setup but no laser activated	Norway	Non-continuous
78	Scolapio et al.	2001	Dysphagia	Endoscopy + balloon cathether	Endoscopy + balloon cathether - not inflated	USA	ES could not be calculated
79	Shaheen et al.	2009	Barrett's dysplasia	Endoscopy + RF treatment	Endoscopy + biopsy + esmoprazole	USA	Non-continuous
80	Steward et al.	2008	Sleep apnea/hypopnea	Palatal implant	Identical implementation device without an implant + a-biotics	USA	ES could not be calculated
81	Stone et al.	2002	Coronary disease	Percutaneous myocardial laser revascularisation	No placebo intervention but patients were blinded during the percutaneous coronary intervention	USA	ES could not be calculated
82	Sutton et al.	1994	Pelvic pain	Laparoscopy + laser ablation	Laparoscopy	UK	Non-continuous
83	Thomsen et al.	1981a	Ménière's disease	Endolymphatic sac decompression	Simple mastoidectomy	Denmark	Non-continuous
84	Toouli et al.	2000	Sphincter of Oddi dysfunction	Endoscopy + sphincterectomy + ERCP	Endoscopy + papillotome introduced into duodenum, noise made but not cut) + ERCP + manometry + morphine/neostigmine provocation test	Australia	Non-continuous
85	van Schie et al.	2000	Diabetic foot	Silicone injections	Saline injection	UK	ES could not be calculated
86	Verheye et al.	2015	Coronary disease	Catheter to the atrium + sinus device	Cathether	USA, Canada, Israel, Italy, Russia	Non-continuous
87	Wei et al.	2012	Urinary stress incontinence	Tape	Sham incisions	USA	Non-continuous
88	Zoumot et al.	2015	Emphysema	Brochoscopy + valve	Bronchoscopy	UK	ES could not be calculated
