# Supplementary material for: The magnitude and temporal changes of response in the placebo arm of surgical randomized controlled trials: a systematic review and meta-analysis
Source: Trials. 2016 Dec 12;17:589. doi: 10.1186/s13063-016-1720-7 (PMC5154040; doi:10.1186/s13063-016-1720-7)
Supplement: Additional file 4: — Included RCTs: list of all surgical RCTs with a placebo arm included in this analysis. (PDF 46 kb) [file 13063_2016_1720_MOESM4_ESM.pdf]

	Study	Year	Condition	Procedure	Placebo intervention	Country	Included in meta-analysis	Blinding	Timing of primary outcome [months]	Cross-over	Outcome	Outcome type	Standard treatment	Randomisation ratio	Number of treatment visits	Comments
1	Abbott et al.	2004	Endometriosis	Laparoscopy + excision	Laparoscopy + staging	UK	Continuous	Patients and assessors	6	C	Pain and QoL	subjective	None	1to1	single event	
2	Arts et al.	2012	Gastroesophageal Reflux Disease	Endoscopy + RF treatment	Endoscopy + setup but no RF	Belgium	Continuous	Patients and assessors	3	C	Distensibility	objective	PPI	1to1	single event	
3	Bradley et al.	2002	Osteoarthritis	Tidal irrigation	Saline injection sub cut. and leg manipulation	USA	Continuous	Patients and assessors	12	N	Pain and function	subjective	Saline sub cutaneously in the placebo group, analgesics	1to1	single event	
4	Buchbinder et al.	2009	Osteoporotic vertebral fracture	Vertebroplasty	Injection of anaesthetic but not cement +cephalosporin	Australia	Continuous	Patients and assessors	3	N	Pain	subjective	Standard medication for osteoporosis	1to1	single event	
5	Buryk et al.	2011	Ankyloglossia	Frenotomy	Sham	USA	Continuous	Patients	12 but no longer fully blinded after 1st day	O	Pain and function	subjective	None	1to1	single event	nipple pain on short form of the McGill Pain Questionnaire SF-MPQ and the Infant Breastfeeding Assessment Tool; IFBAT is reversed
6	Castro et al.	2010	Severe asthma	Bronchoscopy + radiofrequency treatment	Bronchoscopy + placebo procedure	USA, Canda, Netherlands, Israel, Belgium	Continuous	Patients and assessors	6	N	QoL	subjective	Asthma medication	2to1	single event	
7	Corley et al.	2003	Gastroesophageal Reflux Disease	Endoscopy + RF treatment	Endoscopy + setup but no RF delivery	USA	Continuous	Patients and assessors	6	O	Symptoms and QoL	subjective	Antiacids and PPI	1to1	single event	weight in the figure
8	Dowson et al.	2008	Migraine	Patent foramen ovale closure with an implant	Skin incision in the groin + transesophageal US + aspirin and clopidogrel - no heparin	UK	Primary not continuous but ES could be calculated	Patients and assessors	9	N	Pain frequency	subjective	Aspirin and clopidogrel for 90days	1to1	single event	QoL used
9	Eid et al.	2014	Obesity	Endoscopy + gastroplication (StomaphyX)	Endoscopy	USA	Primary not continuous but ES could be calculated	Patients	12	N	Weight loss	objective	None	2to1	single event	
10	Fockens et al.	2010	Gastroesophageal Reflux Disease	Endoscopy + Gatekeeper prothesis	Endoscopy + saline instead of prothesis and instead of antibiotics	USA , Netherlands	Continuous	Patients	6	O	AEs and heartburn symptoms from QoL questionnaire	subjective	PPI and antacids	2to1	resham at 3mo	
11	Freed et al.	2001	Parkinson's disease	Cell transplantation	Incomplete trepanation (dura intact) + PET + MRI + phenytoin - sham-transplantation	USA	Primary not continuous but ES could be calculated	Patients and assessors	12	O	Parkinsonism (UPDRS is secondary)	assessed	Antiparkinsoni an drugs	1to1	single event	there is UPDRS but the primary outcome is change of parkinsonism
12	Freeman et al.	2005	Chronic discogenic pain	Electrothermal therapy	Catheter inserted but not connected + cephazolin +CT	Australia	Continuous	Patients and assessors	6	N	LBOS, SF-36, Pain and function	subjective	Antibiotics, analgesics	2to1	single event	BMI used instead of weight
13	Friedman et al.	2008	Sleep apnea	Palatal implant	Identical implementation device without an implant + a-biotics	USA	Continuous	Patients and assessors	3	O	AHI	objective	Antibiotics, analgesics	1to1	single event	
14	Genco et al.	2006	Obesity	Endoscopy + balloon	Endoscopy but not balloon + diet??	Italy	Continuous	Patients and assessors	3	C	Weight loss	objective	Diet, antiemetics, PPI	1to1	period of time	BMI used instead of weight
15	Gillespie et al.	2010	Sleep apnea	Palatal implant	Identical implementation device without an implant	USA	Continuous	Patients and assessors	1.5	O	CPAP pressure	objective	None	1to1	single event	
16	Gross et al.	2011	Parkinson's disease	Cell transplantation	Scalp incisions and partial-thickness burr holes + MRI - the same duration	USA, Germany	Continuous	Patients and assessors	12	N	Off-state motor UPDRS score	assessed	Antiparkinsoni an drugs	1to1	single event	
17	Guyuron et al.	2009	Migraine	"Deactivation" of trigger points	Exposure of muscles and nervs without changing their integrity	USA	Primary not continuous but ES could be calculated	Patients and assessors	12	O	Pain frequency	subjective	None	2to1	single event	
18	Holmlund et al.	2014	Snoring	Radiofrequency treatment	Setup but no energy delivery	Sweden	Continuous	Patients and assessors	12	N	ESS	subjective	Analgesics	1to1	up to 3 treatments at 4-6week intervals	option to receive multiple treatments
19	Hurwitz et al.	2014	Cancer	Sonication	Setup but no energy delivery	USA, Canada, Israel, Italy, Russia	Continuous	Patients	3	O	Response (pain and medication)	subjective	Analgesics	3to1	single event	worst pain NRS and morphine eqivalent daily dose (MEDD) - MEDD cannot be included as only change from baseline was reported
20	Kallmes et al.	2009	Osteoporotic vertebral fracture	Vertebroplasty	Simulated (audio, sensory, even smell) vertebroplasty - injection of anaesthetic but not cement	USA, UK, Australia	Continuous	Patients and assessors	1	O	Disability and pain	subjective	Analgesics	1to1	single event	
21	Kapural et al.	2013	Chronic discogenic pain	Radiofrequency treatment	No energy delivery	USA	Continuous	Patients and assessors	6	O	Physical function using SF-36 questionnaire	subjective	Back braces, exercises, analgesics, muscle relaxants	1to1	single event	
22	Koutsourelakis et al.	2008	Sleep apnea	Septoplasty	Simulated resection with manipulation of instruments - the same amount of time	Greece	Primary not continuous but ES could be calculated	Patients and assessors	3.5	O	AHI	objective	Saline irigation	1to1	single event	
23	Kvarstein et al.	2009	Lower back pain	Radiofrequency treatment	Sham	Norway	Continuous	Patients and assessors	12	N	Pain	subjective	Analgesics	1to1	single event	
24	Landorf et al.	2013	Plantar callus	Real scalpal callus debridement	Sham scalpal callus debridement	Australia	Continuous	Patients and assessors	1.5	N	Pain	subjective	Usual care	1to1	single event	
25	Larson et al.	1998	Prostatic hyperplasia	Thermoablation	Setup but no energy delivery	USA	Continuous	Patients and assessors	6	O	AUA Score, Qmax, postvoid residual, QoL	subjective	Antibiotic, analgesics, antispasmodic	3to1	single event	Follow-up unblinded after 6 months; "AUA Score" stated first in abstract; Error bars for QoL in figures.
26	Leon et al.	2005	Coronary disease	Percutaneous myocardial laser revascularisation	Setup but no laser procedure	USA	Continuous	Patients and assessors	12	N	Exercise duration	assessed	Antianginal medication	2to1	single event	
27	Lopes et al.	2014	Obsessive-compulsive disorder	Gamma Ventral Capsulotomy	Gamma knife doors remained closed during procedure	Brazil	Continuous	Patients and assessors	12	O	Y-BOCS	subjective	Pharmacologic al standard treatment	1to1	single event	AUA Score, Qmax, postvoid residual, QoL; Y-BOCS stated first in abstract; the use of LOCF imputation could bias your analyses. CGI-I is not usable in meta-analysis as the baseline value is anchored to 0 for everyone.
28	Martinez-Brocca et al.	2007	Obesity	Endoscopy + balloon	Endoscopy	Spain	Continuous	Patients and assessors	4	N	Weight-loss	objective	Diet, antiemetics, PPI	1to1	period of time	Other measures only assessed at 4 weeks; not 4 months.
29	Maurer et al.	2012	Sleep apnea	Palatal implant	Identical implementation device without an implant	Germany	Continuous	Patients and assessors	1.5	N	AHI	objective	None	1to1	single event	
30	McVary et al.	2014	Prostatic hyperplasia	Prostatic Urinary Lift	Rigid cytoscopy	USA, Australia	Continuous	Patients and assessors	3	O	PUL voiding symptoms (IPSS)	subjective	Phosphodieste raze type 5 inhibitors (PDE5i)	2to1	single event	PUL voiding symptoms (IPSS); Peak urinary flow rate (Qmax); Erectile dysfunction (SHIM); Ejaculatory function (MHSQ-EjD); "r" is calculable for all outcomes. I don't understand your "r" sheets.
31	Moseley et al.	2002	Osteoarthritis	Arthroscopy + debridement OR lavage	Skin incision without arthroscopy	USA	Continuous	Patients and assessors	24	N	Pain	subjective	Walking aids, exercises, analgesics	2to1	single event	weight loss as percentage of baseline weight but full data on weight (kg) are available
32	Navada-Castaneda et al.	2003	Dry eye	Lacrimal occlusion with collagen plug	Sham procedure without a plug	Mexico	Continuous	Patients and assessors	2	N	Conjunctivitis symptom score	subjective	Artificial tears	1to1	single event	
33	Nease et al.	2004	Turbinate hypertrophy	Radiofrequency treatment	Sham	USA	Continuous	Patients	6 but cross over at 8weeks	O	VAS obstruction	subjective	Analgesics	1to1	single event	frequency, severity, ability to breathe - frequency mentioned first (as assessed by patients)
34	Olanow et al.	2003	Parkinson's disease	Tissue/cells transplantation	Partial burr holes + a-biotics + cyclosporine + PET	USA	Continuous	Patients and assessors	24	O	UPDRS	assessed	Antiparkinsoni an drugs + cyclosporine	2to1	single event	
35	Pauza et al.	2004	Chronic discogenic pain	Intradiscal electrothermal therapy	Introducing a needle onto the disc (visual and auditory feedback)+discography + CT + prophylactic a-biotics + analgesics + rehabilitation.	USA	Continuous	Patients and assessors	6	N	Pain, disability, SF-36, QoL	subjective	Analgesics, exercise	1to1	single event	
36	Powell et al.	2001	Turbinate hypertrophy	Radiofrequency treatment	Setup but no energy delivery	USA	Continuous	Patients and assessors	1	N	Nasal obstruction VAS	assessed	Analgesics	3to1	single event	
37	Rodriguez et al.	2009	Diabetes mellitus type 2	Endoscopy + bypass liner	Endoscopy + no device	Chile	Continuous	Patients	6	N	HbA1	objective	Diet, metformin or/and sulfonylurea	3to1	period of time	
38	Roehrborn et al.	2013	Prostatic hyperplasia	Cystoscopy + prostatic urethral lift implant	Cystoscopy + simulated procedure	USA, Australia, Canada	Continuous	Patients and assessors	3	O	AUASI	subjective	None	2to1	single event	American Urological Association Sympom Index; follow-up for 12mo but crossover after 3mo
39	Rothstein et al.	2007	Gastroesophageal Reflux Disease	Endoscopy + plication	Endoscopy + setup but device not activated	USA, Germany, Belgium	Continuous	Patients	3	O	QoL	subjective	Antacids and PPI	1to1	single event	
40	Schwartz et al.	2007	Gastroesophageal Reflux Disease	Endoscopy + EndoCinch plication	Endoscopy + setup without needle and thread loaded	Netherlands	Continuous	Patients and assessors	3	O	Heartburn frequency	subjective	Antisecretory drugs	1to1to1	single event	
41	Sihvonen et al.	2013	Degenerative meniscus tear	Arthroscopic partial meniscectomy	Arthroscopy and sham	Finland	Continuous	Patients and assessors	12	O	Pain and function	subjective	Analgesics, exercise	1to1	single event	
42	Silverberg et al.	2008	Alzheimer's disease	Ventriculoperitoneal shunt	Identical shunt but occluded	USA	Continuous	Patients and assessors	9	O	Mattis Dementia Ratings Scale	assessed	Antidementia medication	1to1	period of time	
43	Siproudhis et al.	2007	Faecal incontinence	Elastomer implants	Saline injection	France	Continuous	Patients and assessors	3	N	Success of treatment measured with CC_FI	subjective	Metronidazole, lactulose, paracetamol for 7days	1to1	single event	
44	Stuck et al.	2005	Snoring	Radiofrequency treatment	Device was inserted but not activated	Germany	Continuous	Patients and assessors	3	N	VAS snoring and ESS	assessed	None	1to1	2 sessions 4-6weeks apart	
45	Swank et al.	2003	Abdominal pain	Laparoscopy + adhesiolysis	Laparoscopy	Netherlands	Continuous	Patients and assessors	12	O	Pain and QoL (pain)	subjective	Analgesics	1to1	single event	
46	Thompson et al.	2013	Obesity	Endoscopy + outlet reduction	Sham	USA	Primary not continuous but ES could be calculated	Patients and assessors	6	N	Weight loss	objective	Diet	1to1	single event	
47	Wood et al.	2014	Emphysema	Bronchoscopy + valve	Bronchoscopy + no valve	USA	Continuous	Patients and assessors	6	N	QoL	subjective	Medical management	1to1	single event	QoL measured with St George's Respoiratory Questionnaire
