# Supplementary material for: The magnitude and temporal changes of response in the placebo arm of surgical randomized controlled trials: a systematic review and meta-analysis
Source: Trials. 2016 Dec 12;17:589. doi: 10.1186/s13063-016-1720-7 (PMC5154040; doi:10.1186/s13063-016-1720-7)
Supplement: Additional file 5: — Risk of bias: table summarizing possible list of bias items in each included trial. (PDF 27 kb) [file 13063_2016_1720_MOESM5_ESM.pdf]

Number	Study authors	Publication year	Adequate sequence generation	Allocation concealment	Bias free blinding procedure	Patients blinded	Care-givers blinded	Assessors blinded	Blinding specifically described	All primary outcomes reported	Single primary outcome	Intention-to-treat analysis
1	Abbott et al.	2004	Yes	Yes	Yes	Yes	Yes	Yes	Yes	Yes	No	No
2	Arts et al.	2012	Unclear	Unclear	Unclear	Yes	Unclear	Yes	Yes	Yes	Yes	Yes
3	Bradley et al.	2002	Unclear	Yes	Yes	Yes	Unclear	Yes	Yes	Yes	Yes	Yes
4	Buchbinder et al.	2009	Yes	Yes	Yes	Yes	Yes	Yes	Yes	Yes	Yes	No
5	Buryk et al.	2011	Unclear	Unclear	Yes	Yes	No	No	Yes	Yes	No	Yes
6	Castro et al.	2010	Yes	Unclear	Yes	Yes	Unclear	Yes	Yes	Yes	Yes	Yes
7	Corley et al.	2003	Yes	Yes	Yes	Yes	Unclear	Yes	Yes	Yes	No	No
8	Dowson et al.	2008	Unclear	Yes	Unclear	Yes	Yes	Yes	Unclear	Yes	Yes	Yes
9	Eid et al.	2014	Yes	Yes	Yes	Yes	Unclear	No	Yes	Yes	No	No
10	Fockens et al.	2009	Unclear	Unclear	Yes	Yes	Unclear	No	Yes	Yes	No	No
11	Freed et al.	2001	Unclear	Unclear	Yes	Yes	Yes	Yes	Yes	Yes	Yes	No
12	Freeman et al.	2005	Unclear	Yes	Yes	Yes	Yes	Yes	Yes	Yes	No	No
13	Friedman et al.	2008	Yes	Yes	Yes	Yes	Yes	Yes	Yes	Yes	Yes	Yes
14	Genco et al.	2006	Unclear	Yes	Unclear	Yes	Unclear	Yes	Unclear	Yes	No	Yes
15	Gillespie et al.	2011	Unclear	Yes	Yes	Yes	Yes	Yes	Yes	Yes	Yes	No
16	Gross et al.	2011	Yes	Yes	Yes	Yes	Unclear	Yes	Yes	Yes	Yes	Unclear
17	Guyuron et al.	2009	Unclear	Yes	Yes	Yes	Unclear	Yes	Yes	Yes	No	No
18	Holmlund et al.	2014	Unclear	Yes	Yes	Yes	Unclear	Yes	Yes	Yes	Yes	No
19	Hurwitz et al.	2014	Yes	Unclear	Unclear	Yes	Yes	No	Yes	Yes	Yes	No
20	Kallmes et al.	2009	Yes	Yes	Yes	Yes	Yes	Yes	Yes	Yes	No	Yes
21	Kapural et al.	2013	Yes	Yes	Yes	Yes	Unclear	Yes	Yes	Yes	Yes	Unclear
22	Koutsourelakis et al.	2008	Yes	Unclear	Yes	Yes	Yes	Yes	Yes	Yes	Yes	Yes
23	Kvarstein et al.	2009	Yes	Yes	Unclear	Yes	Yes	Yes	Yes	Yes	Yes	Yes
24	Landorf et al.	2013	Yes	Yes	Yes	Yes	Unclear	Yes	Yes	Yes	Yes	Yes
25	Larson et al.	1998	Unclear	Unclear	Unclear	Yes	Yes	Yes	No	Yes	No	Unclear
26	Leon et al.	2005	Unclear	Unclear	Yes	Yes	Unclear	Yes	Yes	Yes	Yes	Yes
27	Lopes et al.	2014	Yes	Yes	Yes	Yes	Yes	Yes	No	Yes	No	Unclear
28	Martinez-Brocca et al.	2007	Yes	Unclear	Unclear	Yes	Unclear	Yes	No	Yes	Yes	Unclear
29	Maurer et al.	2012	Unclear	Yes	Yes	Yes	Yes	Yes	Yes	Yes	Yes	No
30	McVary et al.	2014	Yes	Unclear	Unclear	Yes	Unclear	Yes	No	Yes	No	Yes
31	Moseley et al.	2002	Yes	Yes	Yes	Yes	Yes	Yes	Yes	Yes	Yes	No
32	Navada-Castaneda et al.	2003	Yes	Yes	Yes	Yes	Yes	Yes	Yes	Yes	Yes	Unclear
33	Nease et al.	2004	Unclear	Unclear	Yes	Yes	No	No	Yes	Yes	No	Yes
34	Olanow et al.	2003	Yes	Unclear	Yes	Yes	Yes	Yes	Yes	Yes	No	Unclear
35	Pauza et al.	2004	Yes	Unclear	Yes	Yes	Yes	Yes	Yes	Yes	No	No
36	Powell et al.	2001	Unclear	Yes	Yes	Yes	Yes	Yes/No	Yes	Yes	Yes	Yes
37	Rodriguez et al.	2009	Unclear	Unclear	Yes	Yes	Unclear	No	Unclear	Yes	Yes	Yes
38	Roehrborn et al.	2013	Yes	Unclear	Yes	Yes	Unclear	Yes	Yes	Yes	Yes	Yes
39	Rothstein et al.	2007	Yes	Yes	Yes	Yes	Unclear	No	Yes	Yes	Yes	Yes
40	Schwartz et al.	2007	Unclear	Yes	Yes	Yes	Unclear	Yes	Yes	Yes	No	Yes
41	Sihvonen et al.	2013	Yes	Unclear	Yes	Yes	Yes	Yes	Yes	Yes	No	Yes
42	Silverberg et al.	2008	Yes	Yes	Yes	Yes	Yes	Yes	Yes	Yes	No	Yes
43	Siproudhis et al.	2007	Unclear	Unclear	Yes	Yes	Unclear	Yes	Yes	Yes	Yes	Yes
44	Stuck et al	2005	Yes	Unclear	Yes	Yes	Unclear	Yes	Yes	Yes	No	No
45	Swank et al.	2003	Yes	Yes	Unclear	Yes	Unclear	Yes	Unclear	Yes	No	Yes
46	Thompson et al.	2013	Yes	Yes	Yes	Yes	Unclear	Yes	Unclear	Yes	Yes	Yes
47	Wood et al.	2014	Unclear	Unclear	Yes	Yes	Unclear	Yes	Yes	Yes	Yes	Yes
